# Comment on “Pregabalin Effect on Acute and Chronic Pain after Cardiac Surgery”

**DOI:** 10.1155/2018/2603865

**Published:** 2018-06-05

**Authors:** Elena Theodorou, Evagelia Bali

**Affiliations:** Department of Anesthesia, Ippokrateio Hospital, Thessaloniki, Greece

We have read with great interest the article entitled “*Clinical Study:* Pregabalin Effect on Acute and Chronic Pain after Cardiac Surgery” by Bouzia et al. [1]. We appreciate their effort for this clinical study, and we would like to make some comments as we believe that there are some flaws in its design and methodology.

First, it is not mentioned when exactly the patients received the single dose of the “study drug” and if there was any delay in the extubation time. In other studies, the results were unstable as time to extubation was significantly longer in the pregabalin group [2, 3] or similar between groups [4, 5].

The visual analogue scale (VAS) and numeric rate scale (NRS) are equally sensitive in assessing acute pain after surgery, and they are both superior to VRS, but the authors chose to use the verbal rating scale (VRS) for assessment of pain intensity, although they finally describe the numeric rate scale. It is remarkable that VRS can be converted to numeric scores for charting and easy comparison, but the authors did not mention or explain this option.

We think that the whole plan of postoperative analgesia has many limitations. First, it is not clear when the patients received the initial dose of morphine (5 mg). Also, they preferred to use a background infusion with the programmable PCA pump and a too long lockout interval combined with a bolus dose of 0.5 mg morphine. Additionally, they provided rescue analgesia with IV morphine if the above scheme with the pump was not sufficient.

It is well known that chronic pain after cardiac surgery is multifactorial and according to the authors with only one dose of pregabalin the patients in the pregabalin groups experienced less severe postoperative pain, used less analgesics, and reported less sleep disturbances without explaining which pain assessment tools they used and if the assessment was done at rest or at stress. Also, it is remarkable that many patients after cardiac surgery may have neuropathic pain characteristics, and it is appropriate to use not only chronic pain assessment tools but also neuropathic pain screening tools such as DN4 [6].

In our opinion, all these limitations may be responsible for their results. More studies and especially well-designed clinical trials are required in order to clarify the optimal dose, the duration of therapy, and the benefits and risks before adopting pregabalin in routine clinical practice and especially in patients undergoing cardiac surgery. Until then, the results may not be as ideal and promising as it sounds.

## References

[1] Bouzia A., Tassoudis V., Karanikolas M. (2017). Pregabalin Effect on Acute and Chronic Pain after Cardiac Surgery. *Anesthesiology Research and Practice*.

[2] Bhojraj S., Agaskar R. D., Kapila S. J., Patil S. K., Behranwala A. A. (2017). Effect of single dose preoperative pregabalin on postoperative pain after cardiac surgery. *Research and Innovation in Anesthesia*.

[3] Pesonen A., Suojaranta-Ylinen R., Hammaren E. (2011). Pregabalin has an opioid-sparing effect in elderly patients after cardiac surgery: a randomized placebo-controlled trial. *British Journal of Anaesthesia*.

[4] Joshi S. S., Jagadeesh A. M. (2013). Efficacy of perioperative pregabalin in acute and chronic post-operative pain after off-pump coronary artery bypass surgery: a randomized, double-blind placebo controlled trial. *Annals of Cardiac Anaesthesia*.

[5] Sundar A. S., Kodali R., Sulaiman S., Karthekeyan R., Vakamudi M., Ravullapalli H. (2012). The effects of preemptive pregabalin on attenuation of stress response to endotracheal intubation and opioid-sparing effect in patients undergoing off-pump coronary artery bypass grafting. *Annals of Cardiac Anaesthesia*.

[6] Jain P. N. (2013). Oral pregabalin holds promise to reduce pain after cardiac surgery. *Annals of Cardiac Anaesthesia*.

